# Recovery Sleep Reverses Impaired Response Inhibition due to Sleep Restriction: Evidence from a Visual Event Related Potentials Study

**DOI:** 10.1371/journal.pone.0142361

**Published:** 2015-12-10

**Authors:** Xiao Jin, Enmao Ye, Jianlin Qi, Lubin Wang, Yu Lei, Pinhong Chen, Guiyun Mi, Feng Zou, Yongcong Shao, Zheng Yang

**Affiliations:** 1 Beijing Institute of Basic Medical Sciences, Beijing, PR China; 2 Cognitive and Mental Health Research Center, Beijing, PR China; 3 Department of Clinical Psychology, Air Force General Hospital, Beijing, PR China; 4 Department of Psychology, The 215th Clinic of 406th Hospital of the Chinese People’s Liberation Army, Dalian, PR China; University of Pennsylvania, UNITED STATES

## Abstract

**Objective:**

To investigate response inhibition after total sleep deprivation (TSD) and the restorative effects of one night of recovery sleep (RS).

**Methods:**

Fourteen healthy male participants performed a visual Go/NoGo task, and electroencephalogram recordings were conducted at five time points: (1) baseline, (2) after 12 h of TSD, (3) after 24 h of TSD, (4) after 36 h of TSD, and (5) following 8 h of RS. The dynamic changes in response inhibition during TSD and after 8 h of RS were investigated by examining the NoGo-N2 and NoGo-P3 event-related potential components.

**Results:**

Compared with baseline, NoGo-P3 amplitudes were decreased, while the NoGo-N2 latency increased along with the awake time prolonged. NoGo anteriorization, which was minimized after 24 h of TSD, progressively decreased with increasing TSD. After 8 h of RS, recoveries of both the NoGo-P3 amplitude and NoGo-N2 latency in the prefrontal cortex were observed compared with the values after 36 h of TSD.

**Conclusion:**

TSD induced a dose-dependent functional decline in the response inhibition of NoGo-N2 and NoGo-P3 on prefrontal cortex activation, and 8 h of RS resulted in recovery or maintenance of the response inhibition. However, it was not restored to baseline levels.

**Limitations:**

Participants were chosen male college students only, thus the findings cannot be generalized to older people and women. Additionally, the sample size was small, and, thus, speculations on the meaning of the results of this study should be cautious. The EEG continuous recording should be employed to monitor the decline of alertness following TSD.

## Introduction

Response inhibition plays an important role in preventing a person from engaging in a preponderant response when that reaction is not appropriate, which is very crucial for flexible performance and goal-oriented behaviors that are based on the current environment [1]. Previous studies have indicated that response inhibition involves two cognitive components: attention to incoming stimuli and prevention of automatic responses [2]. According to these studies, the neural mechanism of response inhibition can be measured by a Go/NoGo task, which is an experimental paradigm widely used in executive brain function research. In the Go/NoGo task, participants are asked to respond when frequent Go stimuli are presented and to withhold their responses to infrequent NoGo stimuli. Compared with the Go trials, two event-related potential (ERP) components are observed at prefronto-central electrodes during NoGo trials. One is the N2 component, which is a negative deflection that occurs around 140–300 ms, and the other is the P3 component, which is a positive deflection that occurs around 300–600 ms [3, 4]. Neuroimaging studies have suggested that the No-Go stimuli-elicited anterior-driven N2 and P3 components are generated from the anterior cingulate cortex, and these components reflect stimuli detection and response inhibition, respectively [5], which are higher-order cognitive processes that specifically rely on the prefrontal cortex.

Response inhibition can be adversely affected by sleep loss [6–8]. For example, 24 h of total sleep deprivation (TSD) results in impairments in both error detection and error remediation and a failure to avoid making the errors again [9]. Several studies have revealed that TSD impairs inhibition control and increases impulsive behaviors in response inhibition tasks [8, 10]. If the TSD is not long enough, the NoGo-N2 amplitude will not change because of the cerebral compensatory responses to the increased monitoring demands [9]. However, when the TSD is prolonged to 43 h or longer, the amplitudes of NoGo-N2 and NoGo-P3 are significantly decreased compared with baseline levels [11]. Furthermore, the prolonged P3 latencies and reduced P3 amplitudes during extended wakefulness suggest severe impairment of the response inhibition due to the long periods of sustained sleep restriction.

Recovery sleep (RS) is needed to improve inhibition performance after a long TSD [12]. Several studies have shown that the electrophysiological mechanisms for improving cognitive brain function during RS might involve increased delta power in the prefrontal cortex [12, 13]. Drummond and colleagues have suggested that the impaired inhibition and automatic responses that are induced by TSD can be completely remedied back to baseline level by one night of RS [14]. However, Wu and colleagues have indicated that even though RS does improve human performance, one night of sleep does not seem sufficient for full recovery of the impaired prefrontal function [15]. Whether 8 h of RS followed by a long period of wakefulness will result in full recovery of the response inhibition remains to be elucidated. Furthermore, the neural mechanisms underlying the recovery of the function of the prefrontal cortex and response inhibition are still unknown.

In the present study, a visual Go/NoGo task was employed with simultaneous electroencephalogram (EEG) recordings at 5 time points during the 36 h of TSD and after RS [0 h of TSD (Baseline), 12 h of TSD, 24 h of TSD, 36 h of TSD, and 8 h of RS]. It evaluated the following: (1) the dynamic changes of the N2 and P3 ERP components in the visual Go/No-Go task during the TSD and (2) the effects of 8 h of RS on response inhibition after 36 h of TSD. We assumed that the N2 and P3 components in the No-Go trials would attenuate with the prolonged TSD period and that the impaired response inhibition would be reversed after one night of RS.

## Materials and Methods

### Participants

Fourteen healthy male adults (age: 25.9 ± 2.3; range, 18–28) were recruited from Beijing Normal University as paid volunteers. None of the participants had ever been involved in a psychophysiological experiment, and they all provided written informed consent prior to the start of the experiment. The general exclusion criteria were diseases of the central and peripheral nervous system, encephalic trauma, cardiovascular diseases and/or hypertension, cataracts and/or glaucoma, pulmonary problems, audiological problems, and alcohol or drug abuse. The participants did not smoke or drink alcohol and had normal or corrected-to-normal vision. All of the participants had normal intelligence (Raven test, IQ>100) and T scores on the General Symptom Index less than 60 (the general population has T scores over 63) [16, 17]. At the screening stage, the Pittsburgh sleep quality index (PSQI) [18] was employed to rule out the participants with early rise or late sleep habits. The experiment was conducted in the Basic Aerospace Institute (Beijing, China) with nursing staff attending all the time. The subjects always had a partner with them during the study so they could keep each other awake through the night during continuous behavioral monitoring, and subjective sleepiness was assessed every 4 h. The experimental protocol was approved by the Ethics Committee of the Beijing Institute of Basic Medical Science.

### Experimental design

**Fig 1:**
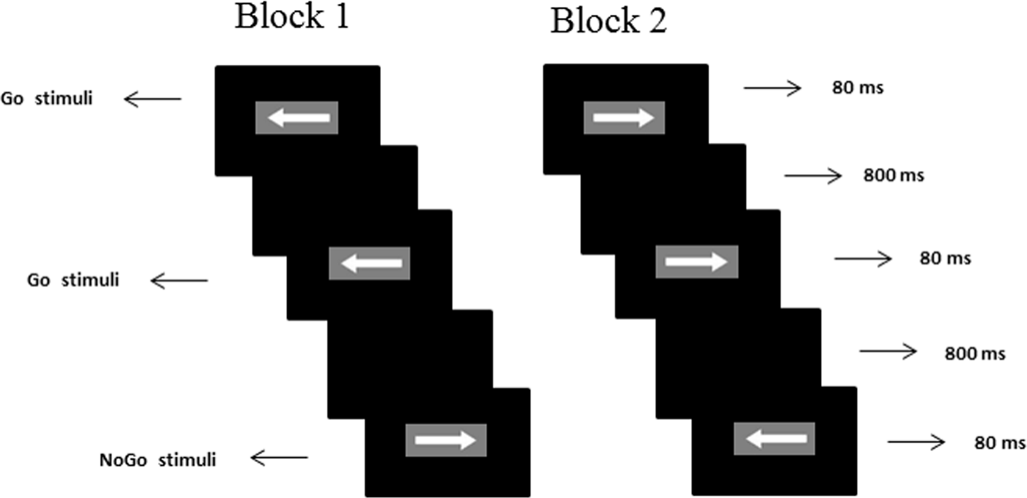
Go/NoGo stimuli type and temporal sequence of events in the task trials that consisted of two types of stimuli in the protocol. In block 1, the participants were supposed to respond to the left arrow and withhold their response to the right arrow. In block 2, the response pattern was reversed. Each stimulus was presented with a duration of 80 ms and an interstimulus interval of 1,000 ms. The time window of the responses was 800 ms.

The visual Go/NoGo task was presented on a screen with a resolution of 1,280 × 768 pixels. The Go/NoGo task consisted of two arrow types (left and right, 78 × 18 pixels each, white visual stimuli with gray background) that were presented in a serial random pattern. Each trial began with the appearance of a + at the center of the screen. As shown in Fig 1, stimuli were presented for 80 ms with an inter-stimulus interval of 1,000 ms. Time window for the responses was set to 800 ms.

The task was presented in two parallel blocks of 200 trials each (33% were No-Go trials and 67% were Go trials), which lasted for a total of 400 s. In the first block, the participants were instructed to respond to the left arrow and withhold response to the right arrow. In this case, the left arrow represented the Go stimulus, while the right arrow represented the No-Go stimulus. In the second block, the right arrow represented the Go stimulus, while the left arrow represented the NoGo stimulus. The participants were instructed to press a button when the Go stimulus appeared and to withhold their response when the NoGo stimulus appeared. In addition, the participants were told to press the key as fast as possible while maintaining a high level of accuracy.

The experiment was conducted in a quiet, magnetically shielded, and darkened room. The visual Go/NoGo task was performed with simultaneous EEG recording. The participants passively viewed the 21-in display that was 75 cm in front of them, and they were asked to maintain their gaze at the fixation circle throughout the experiment (visual angle, 3.29° × 1.76°). A training session was performed to ensure that the participants understood the task. All of the participants had hit rates that were 90% or above before the formal test.

**Fig 2:**
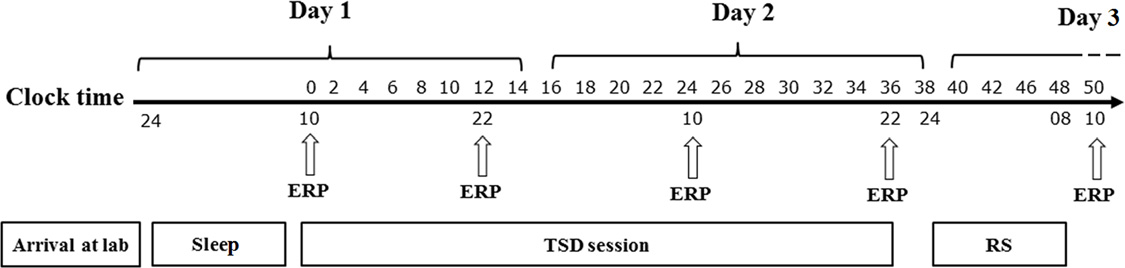
Schematic diagram of the experimental design. TSD started after a routine nocturnal sleep period. The clock time axis represents the course of TSD and RS. TSD started at 10:00 pm at Day 1. The subjects needed to keep awaking for 36 h. Each arrow below the clock time axis represents the time points performed the Go/No-go task and EEG recording synchronously. The interval of the tasks was 12 hours during the TSD session. At the time point of 24:00 pm, Day 2, all participants accepted an 8-h recovery sleep.

After one week of normal sleep, the subjects visited the laboratory for the formal experiment. They had been required to maintain a regular sleep schedule of 8 h per night at least one week before the study. And all participants were asked to sign a sleep diary before the experiment to ensure the sleep quality. Central inhibition or stimulant drugs were forbidden during the entire experiment in the laboratory. The TSD started at 10:00 am the day (Day 1) after a routine sleep, and all participants needed to complete 4 times of Go/NoGo tasks within 12-h intervals. After 36 h of TSD, the participants received 8 h RS (Day 2 to Day 3), and the participants then performed the last Go/NoGo task (Fig 2). During the TSD, the Visual Analogue Scales (VAS), a self-assessment scale to evaluate subjective emotional state [19], was applied to assess the participants’ subjective sleepiness for every 4 h. The two partner participants performed the experiments at the same time. The nursing staff accompanied the participants all of the time in order to prevent the participants from falling asleep by observation and remind them throughout TSD session.

### EEG recordings

Continuous EEG recordings were made with a 32-channel SynAmps2 amplifier (Compumedics Neuroscan, Charlotte, NC, USA). The participants wore an Ag/AgCl electrode cap (Quik-Cap, Compumedics Neuroscan) that had electrodes at the 32 sites specified by the International 10–20 system. EEG recordings were sampled at 1,000 Hz, and the impedances were maintained below 5 kΩ for all of the channels. The bilateral mastoids (M1 and M2) were used for reference, and the forehead was used as a ground. Four additional electrodes were placed above and below the left eye in order to perform bipolar vertical electro-oculogram recordings, and horizontal electro-oculogram recordings were taken from the outer canthi of both eyes.

### Behavioral and ERP statistical analyses

**Fig 3:**
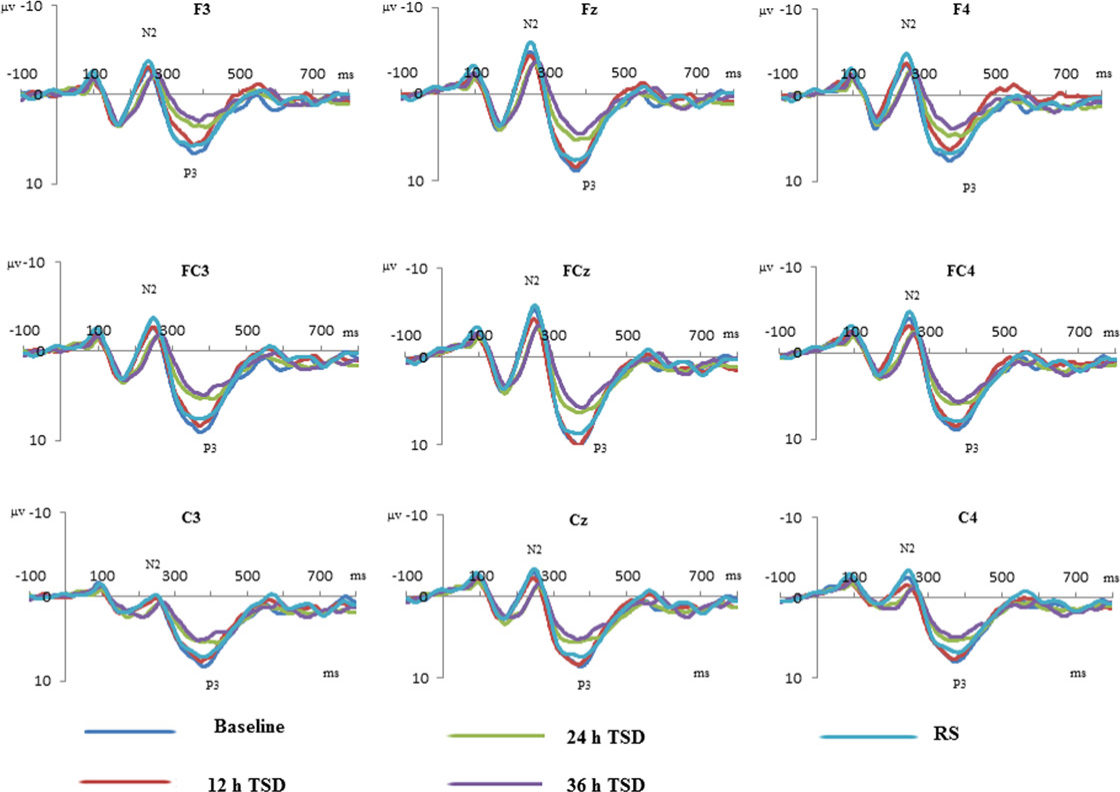
Grand-average event-related potential waveforms at F3, FC3, C3, Fz, FCz, Cz, F4, FC4, and C4 for NoGo trials in different time points. The color waveforms represents respectively the baseline, after 12-h of total sleep deprivation (TSD), after 24-h of TSD, after 36-h of TSD and after 8-h recovery sleep.

All of the EEG preprocessing was performed with the Compumedics NeuroScan software package (Version 4.3). For all of the participants, band-pass filters were applied between 0.05 Hz to 100 Hz with a modest 12/24-dB slope, including a 50-Hz notch filter that was applied to remove mains power noise. Ocular artifacts were estimated and subtracted with a time-domain regression analysis [20]. The continuous EEG recordings were segmented into epochs starting 100 ms before the onset of the stimulus and continuing until 800 ms after stimulus onset. The baseline was corrected to mean amplitude of 100 ms before the stimulus. The trials in which the voltage exceeded ±100 μV were rejected automatically by the system. The components were calculated with only the corrected responses, and the main ERP components were averaged. The grand-averaged ERPs were displayed graphically in order to identify the major peaks. The NoGo-N2 and NoGo-P3 components were defined as the maximum negative or positive peaks within the 150–300-ms and 300–600-ms latency windows, respectively, after the onset of each stimulus. According to No-Go anteriorization [21], only the components that were recorded at the F3, Fz, F4, FC3, FCz, FC4, C3, Cz, and C4 electrodes were included for further analysis (Fig 3).

**Fig 4:**
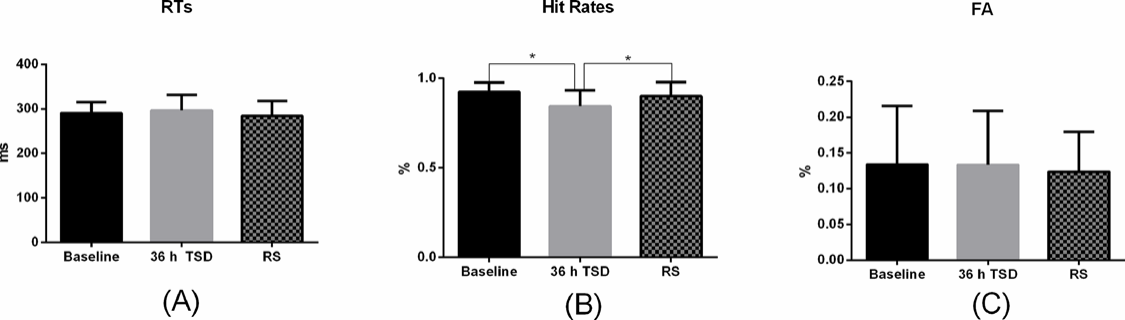
The differences of RT and Hit Rates in Go trials and FA in No-Go trials between Baseline, 36 h TSD and 8 h RS. RTs: The Reaction Time, FA: False Alarms, Baseline: before total sleep deprivation session, 36 h TSD: after 36 hours of total sleep deprivation, 8 h RS: after 8 hours of recovery sleep. (A): The mean of RTs in Baseline, 36 h TSD and 8 h RS. (B): The mean of Hit Rate in Baseline, 36 h TSD and 8 h RS. (C): The mean of FA in Baseline, 36 h TSD and 8 h RS. The error bars represent standard deviation. *: p<0.05.

The data for two subjects were excluded from the study due to technical errors. Finally, the data for 12 subjects were included in the following statistical analysis. All of the analyses were conducted with PASW Statistics 18.0 for Windows, as shown in Table 1. The outcome variables for behavior included the average reaction time (RT), the hit rates on the Go trials, and the percentage of false alarms (FA). The effect of RS on behavior indexes was presented in Fig 4.

**Table 1:** The Reaction Time and Hit Rates in Go trials and False Alarms in No-Go trials.

Behavioral variables	Baseline	Total Sleep Deprivation		
	0 h	12 h	24 h	36 h
**RTs (Go)**	290.63(27.75)	285.46(28.6)	299.45(32.15)	296.47(34.63)
**Hit Rates (Go)**	0.924(0.016)	0.919(0.026)	0.817(0.035)	0.845(0.025)
**FA (NoGo)**	0.134(0.024)	0.128(0.019)	0.144(0.022)	0.133(0.022)

h, hour; RT, reaction time; FA, false alarm.

The data are presented as mean (standard deviation).

The time unit for reaction time (RTs) is millisecond (ms).

**Fig 5:**
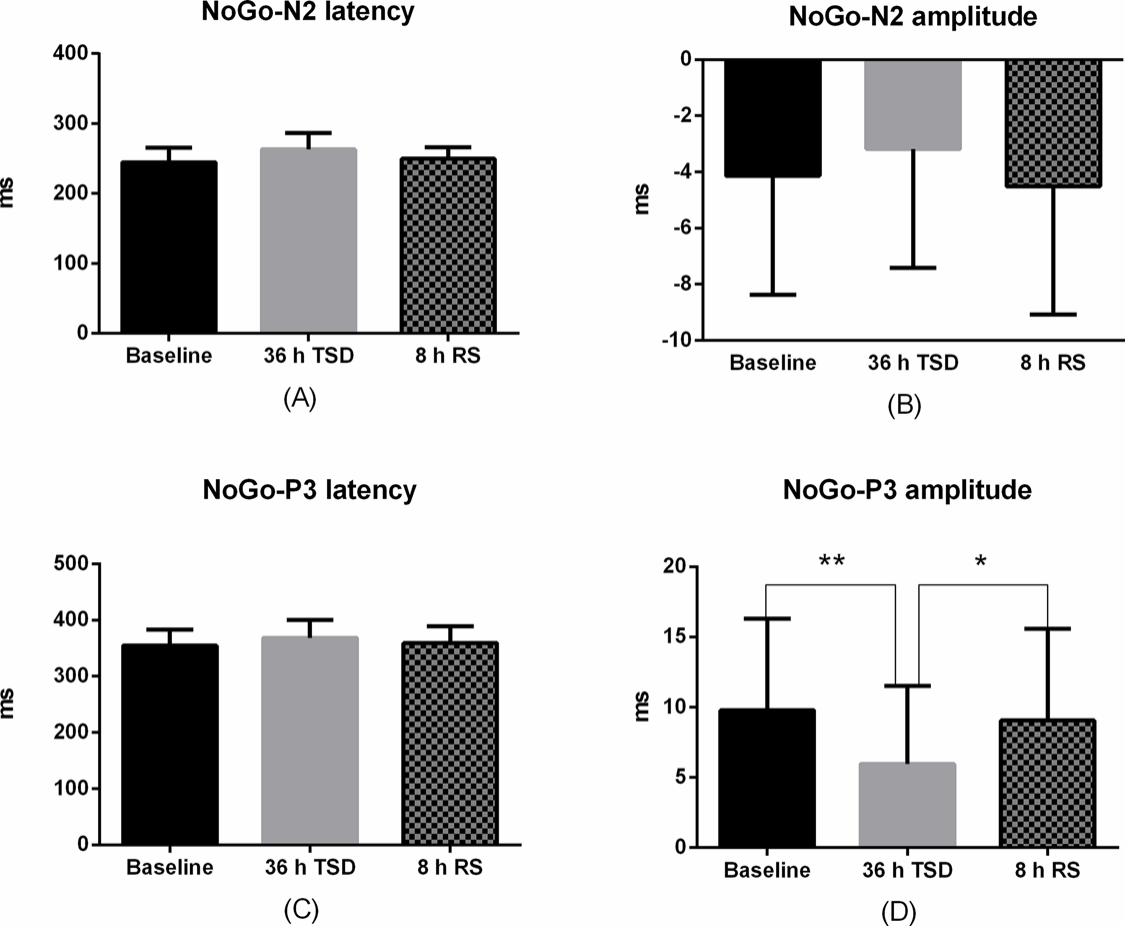
ERP components differences between Baseline, 36 h TSD and 8 h RS. Baseline: before total sleep deprivation session, 36 h TSD: after 36 hours of total sleep deprivation, 8 h RS: after 8 hours of recovery sleep. (A) The mean of NoGo-N2 latency in Baseline, 36 h TSD and 8 h RS. (B) The mean of NoGo-N2 amplitude in Baseline, 36 h TSD and 8 h RS. (C) The mean of NoGo-P3 latency in Baseline, 36 h TSD and 8 h RS. (D) NoGo-P3 amplitude in Baseline, 36 h TSD and 8 h RS. The error bars represent standard deviation. *: p<0.05 (uncorrected), **p<0.025 (corrected), corrected multiple comparison analysis.

The N2 and P3 amplitudes and latencies that were elicited by NoGo trials at the nine electrode sites are presented in Fig 3. The standard deviations of the N2 and P3 components during TSD are shown in Tables 2 and 3. The effect of RS on ERP components was presented in Fig 5.

**Table 2:** Average NoGo-N2 Latencies and Amplitudes at F3, Fz, F4, FC3, FCz, FC4, C3, CZ, and C4 after Different Lengths of Total Sleep Deprivation.

	Baseline	Total Sleep deprivation		
	0 h	12 h	24 h	36 h
**Latency(ms)**				
**F3**	244.25(27.56)	245.17(22.57)	263.67(26.65)	266.25(27.11)
**Fz**	248.25(16.8)	246.25(19.11)	265.92(17.83)	266.42(23.82)
**F4**	247.17(23.95)	246.58(20.66)	262.75(22.86)	267.08(22.06)
**FC3**	241.92(21.74)	244.00(22.34)	258.08(27.93)	265.75(27.61)
**FCz**	247.17(16.49)	245.5(18.60)	258.67(23.40)	261.25(20.02)
**FC4**	244.83(17.63)	243.92(20.62)	260.08(19.51)	265.00(23.70)
**C3**	244.83(24.70)	242.25(28.05)	262.25(34.47)	261.08(27.28)
**Cz**	243.08(20.50)	241.67(21.49)	255.08(23.01)	258.83(20.92)
**C4**	240.92(21.35)	240.08(22.27)	257.25(16.56)	257.25(16.56)
**Amplitude(μV)**				
**F3**	-3.70(4.25)	-3.70(3.24)	-2.99(4.17)	-3.51(3.76)
**Fz**	-5.66(4.29)	-5.14(3.01)	-4.42(4.59)	-4.99(4.28)
**F4**	-4.44(4.04)	-4.64(4.15)	-3.67(4.28)	-4.03(4.15)
**FC3**	-3.73(3.90)	-3.49(3.05)	-2.58(4.21)	-3.06(3.95)
**FCz**	-6.25(4.47)	-5.02(4.09)	-3.86(5.00)	-4.93(4.96)
**FC4**	-4.78(4.32)	-3.97(3.62)	-3.00(4.64)	-3.27(4.24)
**C3**	-1.23(3.68)	-0.84(3.51)	-0.24(3.43)	-0.36(3.33)
**CZ**	-3.93(4.77)	-3.07(4.76)	-1.87(4.97)	-2.66(4.69)
**C4**	-3.50(3.60)	-2.28(3.57)	-1.65(4.03)	-1.78(3.82)

The data are presented as mean (standard deviation).

**Table 3:** Average NoGo-P3 Latencies and Amplitudes at F3, Fz, F4, FC3, FCz, FC4, C3, CZ, and C4 after Different Lengths of Total Sleep Deprivation.

	Baseline	Total Sleep deprivation		
	0 h	12h	24h	36h
**Latency(ms)**				
**F3**	356.50(32.01)	355.33(28.05)	364.92(40.87)	368.25(33.53)
**Fz**	354.42(27.67)	356.75(25.70)	364.58(39.99)	368.83(34.22)
**F4**	356.92(31.69)	354.67(27.47)	361.00(42.03)	469.83(34.22)
**FC3**	352.33(27.99)	358.75(25.27)	367.00(39.54)	374.17(29.21)
**FCz**	353.00(25.26)	355.42(23.71)	365.00(38.30)	365.33(27.19)
**FC4**	356.33(27.19)	356.75(24.32)	363.08(39.24)	367.50(33.76)
**C3**	356.42(32.49)	364.08(23.15)	369.25(39.17)	368.33(32.73)
**CZ**	352.67(28.84)	355.75(29.42)	355.75(29.42)	365.92(38.55)
**C4**	356.08(27.07)	358.67(26.52)	367.92(40.25)	368.08(33.82)
**Amplitude(μV)**				
**F3**	8.12 (4.58)	6.47(3.80)	5.47(5.73)	3.97(4.23)
**Fz**	10.34(7.38)	9.47(6.11)	7.82(7.91)	5.76(6.00)
**F4**	9.21(5.90)	7.35(4.89)	6.89(6.48)	5.16(5.19)
**FC3**	10.43(6.23)	9.12(5.31)	7.37(7.02)	5.84(5.14)
**FCz**	11.74(9.22)	11.16(7.64)	8.97(9.07)	7.28(7.49)
**FC4**	10.36(7.03)	9.21(5.72)	7.84(7.40)	6.94(6.21)
**C3**	8.94(5.48)	8.17(4.58)	6.79(5.47)	5.65(4.39)
**CZ**	9.67(6.82)	9.6(6.82)	7.72(7.53)	6.84(6.63)
**C4**	8.79(5.85)	8.18(4.37)	6.69(6.01)	6.13(5.10)

The data are presented as mean (standard deviation).

In order to examine the effects of TSD on response inhibition, a repeated measures analysis of variance (ANOVA) was employed to analyze behavioral performance at the selected time points during TSD. The baseline, 12-h TSD, 24-h TSD, and 36-h TSD measures were the within-subject factors. For the NoGo-N2 or NoGo-P3 components during TSD, a three-way repeated ANOVA was conducted with functional cortex (prefrontal, prefronto-central, and central), site (Left, Midline, and Right), and the TSD time points (0 h, 12 h, 24 h, and 36 h) as the within-subject factors and the N2 and P3 amplitudes and latencies as the dependent variables. In further statistical analyses, we explored the effects of RS on the response inhibition in the different conditions, including baseline, 36-h TSD, and RS. In addition, we also employed a one-way ANOVA to separately analyze the condition effects (Baseline, 36-h TSD, and RS) on the behavioral index. The statistical significant value was set at 0.05 for behavioral index. We performed a three-way repeated ANOVA with functional cortex (prefrontal, prefronto-central, and central), site (Left, Midline, and Right), and the TSD time points (Baseline, 36-h TSD, and RS) on the NoGo-N2 or NoGo-P3 components during TSD. Greenhouse-Geisser corrections was applied if necessary. Corrected multiple comparison was applied to eliminate the Bonferroni effect. The *p* values less than 0.016 or 0.025 were considered statistically significant for TSD effect and RS effect comparison, respectively.

## Results

### Behavioral performance

The result of VAS showed that the participants were sleepier (t = -4.45, *p* = 0.001) after 36 h than the baseline level. The RTs of Go-trials, which reflected a prolonged response time window, did not show significant main effect of time during TSD [F (3, 44) = 1.824, *p* = 0.162]. The hit rates in the Go trials showed a significant main effect of time during TSD [F (3, 44) = 3.45, *p* = 0.028]. Further post-hoc analysis revealed that the hit rates in the Go trials after 36 h of TSD were significantly lower than those at baseline (mean difference = 0.079, *p* = 0.014). The FA rates in the No-Go trials did not differ [F (3, 44) = 0.288, *p* = 0.833] under the TSD session. A significant main effect of condition on the hit rates in the Go trials was found [F (2, 33) =, *p* = 0.008]. Further post-hoc analysis that after 36-h TSD, hit rates were decreased significantly (mean difference = -0.079, *p* = 0.014) compared with the baseline. However, the hit rates of the Go trials were increased significantly after RS compared with 36-h TSD (mean difference = 0.056, *p* = 0.02). No significant difference was found for the hit rates on Go trials between the baseline and RS (Fig 4). Furthermore, no main effects of condition were found in RTs of the Go trails and FA rates of the No-Go trials.

### NoGo-N2

The means and standard deviations of the NoGo-N2 amplitudes and latencies at the F3, FZ, F4, FC3, FCZ, FC4, C3, CZ, and C4 electrodes during TSD are listed in Table 2. There was a significant main effect of time on the NoGo-N2 latency [F (3, 44) = 8.954, *p* = 0.000] during TSD. Adjusted multiple comparisons showed that there is no difference between the baseline and 12 h TSD for the NoGo-N2 latency. After 24 h of TSD, the NoGo-N2 latency was increased when compared to 12 h TSD (mean difference = 16.481, *p* = 0.010). Additionally, the NoGo-N2 latency was increased continuously after 36 h TSD and significantly longer than that of 12 h TSD (mean difference = 19.728, *p* = 0.007). No significant main effects were found for functional cortex or site on NoGo-N2 latency during TSD.

Significant main effects of functional cortex and site on NoGo-N2 amplitude were found [F (2, 33) = 16.130, *p* = 0.000; F (2, 33) = 13.957, *p* = 0.002, respectively] under condition of TSD. During TSD, the maximum amplitude of NoGo-N2 appeared at prefrontal cortex (Fig 3). The NoGo-N2 amplitude in the central cortex was smaller than that in the prefrontal and prefronto-central cortex (F3/Fz/F4 vs. C3/Cz/C4: mean difference = -2.290, *p* = 0.014; FC3/FCz/FC4 vs. C3/Cz/C4: mean difference = -2.044, *p* = 0.000). Moreover, the NoGo-N2 amplitudes in the middle electrodes were more negative than bilateral areas (Table 2). The left electrode site reached the statistical significance when compared with the middle and the right electrode site (Left vs. Midline: mean difference = 1.864, *p* = 0.002; Left vs Right: mean difference = 0.965, *p* = 0.008). There is no significant difference between the right site and the middle site (*p* < 0.016). No other interaction effects were found during the TSD (*p* < 0.016).

In the analysis of recovery sleep effects on the ERP components, we found a significant main effect of condition (baseline, 36 h of TSD, and RS) on NoGo-N2 latency [F (2, 33) = 7.66, *p* = 0.003]. The corrected multiple comparison analysis did not show any significant difference between baseline, 36 h of TSD and RS. No other significant main effect or interaction effects were found on NoGo-N2 latency among different conditions (*p* < 0.025). For NoGo-N2 amplitude, main effects were found for functional area [F (2, 33) = 11.023, *p* = 0.004] and site [F (2, 33) = 22.745, *p* = 0.000], but the main effect of condition was not observed. The corrected multiple comparison analysis showed that NoGo-N2 amplitudes in the prefronto-central area were larger than the amplitudes in central regions (FC3/FCz/FC4 vs. C3/Cz/C4: mean difference = -2.163, *p* = 0.001; Fig 3). No significant difference was found for NoGo-N2 amplitudes between the prefrontal cortex and the prefronto-central area. In addition, the NoGo-N2 amplitudes in the midline were more negative than those from the bilateral electrodes (Midline vs. Left: t = -2.234, *p* = 0.001; Midline vs. Right: t = -1.076, *p* = 0.012; Fig 3). No other interaction effects reached statistical significance (*p* < 0.025).

### NoGo-P3

The means and standard deviations of the NoGo-P3 amplitudes and latencies at the F3, FZ, F4, FC3, FCZ, FC4, C3, CZ, and C4 electrodes are listed in Table 3. Significant main effect of time on NoGo-P3 latency was found during TSD with uncorrected Bonferroni effect [F (3, 44) = 3.005, *p* = 0.044]. No other significant interaction effects reached statistical significance (*p* < 0.016) in TSD session.

For NoGo-P3 amplitude, main effects were observed for time [F (3, 44) = 8.160, *p* = 0.000] during TSD. The NoGo-P3 amplitudes were reduced remarkably after 36 h TSD when compared with baseline (t = 3.828, *p* = 0.015) as shown in Table 3. Although the NoGo-P3 amplitudes decreased and exhibited a dose-dependent pattern along with prolonged time of TSD (Table 2), the difference did not reach statistical significance (*p* < 0.016). No other interaction effects reached statistical significance for NoGo-P3 amplitudes under the TSD session (*p* < 0.016).

In the analysis of recovery sleep effect on NoGo-P3 components, no main effect was found for the different condition (*p* < 0.025) on the NoGo-P3 latency. For NoGo-P3 amplitude, significant main effects of condition were observed [F (2, 33) = 8.182, *p* = 0.002]. The corrected multiple comparison analysis showed a reduced NoGo-P3 amplitude after 36 h of TSD when compared with the baseline (mean difference = 3.828, *p* = 0.007). Although NoGo-P3 amplitude showed increased tendency after 8 h RS compared with baseline, but the difference did not reach statistical significance (*p* < 0.025) (Fig 5). No other significant interaction effects reached statistical significance (*p* < 0.016) in TSD session.

### The correlation between the behavioral performances change and the ERP component change

For analyzing the correlation between the behavioral performance and the ERP components change altered, we use Pearson correlation analysis to calculate the correlation coefficient of the changed behavioral performances and ERP component. The variables of behavioral performance include RTs and Hit Rates for Go trials, and FA for the NoGo trials. The ERP components include N2 latencies, N2 aptitude, P3 latencies and P3 aptitude in all electrodes sites. The Pearson correlation showed that positive correlations were found between the Hit Rates for Go trials and P3-aptitude in FC4 (r = 0.611, p = 0.035), Fz (r = 0.609, p = 0.035), FCz (r = 0.697, p = 0.012), C4 (r = 0.613, p = 0.034), Cz (r = 0.654, p = 0.021). However, other correlation between the behavioral performances change and the ERP component change had not been found

## Discussion

Here we reported an investigation of the effects of TSD and RS on response inhibition with a simultaneous visual Go/NoGo task and EEG recordings. We recorded both behavioral and ERP indexes at 5 different time points in order to observe how response inhibition was altered during TSD and to what extent one night of normal sleep recovered the impaired executive brain function. In the behavioral analyses, we observed that the hit rates in the Go trials decreased markedly after TSD. However, FA, which reflected inhibition function, seemed less affected during TSD. Because response inhibition is a complex cognitive process, the behavioral task that was used in the present study may not have been sensitive enough to accurately reflect the temporal characteristics of response inhibition.

Because the circadian rhythm expresses distinct individual difference, we adopted the EEG data from the same time point in the same experimental schedule to ensure the subjects with the similar level of alertness. Circadian rhythm is related to the behavior performance along with the prolonged TSD [22]. Babkoff found that work capability decline fluctuated, and related to 24 hours of temperature curve of circadian rhythm, obviously at 2:00 am to 6:00 am. Neurobehavioral functions are affected by endogenous circadian rhythms, which including physiological alertness and cognitive performance [23]. Previous researches had been proved that cognitive function is sensitive to TSD [24, 25], so it would be best to evaluate response inhibition ability avoiding decline phase of circadian rhythm. In this study, 10:00 am and 10:00 pm was selected to performed experiment avoiding the effect of circadian rhythms on the cognitive function [26]. Due to complex circadian rhythms effect varies across individuals, the effects could not be total excluded during the TSD experiment [27, 28]. In line with our hypothesis, we observed a delay in the peak latencies tendency and a significant reduction in the amplitudes of NoGo-P3 during TSD. These results were consistent with several previous studies that reported the latency of P3 is positively related to sleepiness. Moreover, these studies suggested that extended wakefulness is related to the prolonged latency and reduced amplitude of the P3 component [21, 29, 30]. Because the P3 component is thought to reflect attention resource deployment and the P3 latency is thought to reflect the time window for stimulus categorization and evaluation, the increase in the P3 latency tendency that occurred during the prolonged TSD suggested that more time was needed for attention resource allocation after TSD. The decreased amplitudes of the NoGo-P3 potentials after 24 h of TSD, which may reflect the participants’ reduced concentration, also shows reduced discrimination to the target stimuli [22]. The prolonged latency tendency of P3 may indicate that subjects are experiencing difficulty allocating attentional resources to detect No-Go stimuli after TSD.

The N2 and P3 components of ERPs are related to the distinct sub-processes that occur in the response inhibition function [31]. In our study, the NoGo-N2 component only showed prolonged latency and did not show significant changes in amplitude. In accordance with the findings of Zhang’s study, the prolonged NoGo-N2 latency may reflect increased reaction time after a long period of sleep restriction [32]. These findings suggest that the TSD induced a difficulty in reducing the impulse to an inappropriate response. In addition, it could be the reduced speed of the response-selection processes that are required in the Go/NoGo task. The finding that N2 amplitude was not significantly altered in the NoGo trials may have been due to a cerebral compensatory response that involves an increase in the monitoring demand of the response selection from the TSD [9]. These results may indicate that a compensation mechanism exists for the impaired response inhibition, and, therefore, no significantly decreased N2 amplitudes were observed in this study. Another interpretation of these results is that the NoGo-N2 component covaries with the magnitude of the FA rates because FA rates increase after sleep deprivation [33].

For ERP component changes in NoGo trials, we had interestingly results of functional effects.

Consistent with previous studies, the NoGo-N2 amplitude showed negative tendency at the prefrontal and prefronto-central area than central functional area with TSD influence. Falkenstein and colleagues had reported that NoGo anteriorization both in NoGo-P3 and NoGo-N2 amplitude, most obviously for NoGo-N2 amplitude at the frontal area. Also they had observed that NoGo-N2 amplitude decrease steadily from electrode Fz to Oz [34]. The possible explanation for this phenomenon is that NoGo-N2 peak amplitude was located at the right fronto-central site, and the generating source of NoGo-P3 localized in left orbitofrontal cortex [35]. We did not found the effects of laterality of NoGo-P3 and NoGo-N2 amplitudes, which are maximum on central electrodes such as Fz, FCz and Cz [36, 37].

After 8 h of RS, although we observed an increase in NoGo-P3 amplitude compared with this value after 36 h of TSD, but the increase did not reach the significant statistically. Still, the recovery tendency for NoGo-P3 amplitude existed [Fig 4(D)]. Current results support the view that taking 1 to 8-h naps enhances performance and alertness during continuous operations [32, 38]. Interestingly, the increased latency of NoGo-N2 and NoGo-P3 that was induced by TSD was not significantly improved by one night of RS. Several studies have contended that an intense rebound occurs following sleep after a TSD [39, 40]. The rebound involved many changes, such as response inhibition function decline, increased delta power, decreased sigma power, and increased sleep time and efficiency [39, 41, 42]. Cajochen and colleagues have reported that the mechanism for improving function may be related to a dominant delta power throughout the frontal cortex that is induced by RS [43]. However, one night of RS after a long period of TSD may not be enough for full recovery of prefrontal brain function [15]. Our results of non-recovery of the NoGo-P3 amplitude provide evidence that the function for withholding inappropriate responses did not fully recover, as we speculated. Execution is a function that largely depends on activity in the prefrontal cortex [44]. In the present results, we observed obviously large N2 amplitude that was induced by No-Go stimuli in the prefrontal and prefronto-central area, which indicated the activation of prefrontal function due to the TSD. However, No-Go anteriorization still appeared in the NoGo-N2 component during the TSD, which indicated that the early stage of cognitive processing was less affected. Because the NoGo-N2 component is related to the Go stimuli [44, 45], these findings suggest that simple cognitive responses are less affected by TSD and can recover easily following RS, while higher cognitive brain function that is related to the NoGo-P3 is difficult to reverse. From the results for the NoGo-P3 component, we speculate that although the participants applied more attentional resources to the current task, their reaction speed to recognize which stimuli required the withholding of a response did not seem to improve. One possible explanation is that the improvements in executive function by RS were mainly reflected in the restoration of alertness, which allowed the distribution of more attentional resources to the current task of stimulation recognition and classification.

### Limitations

Our study had several limitations. First, we only examined male college students in this study. Therefore, these results are not generalizable to older or female subjects. Second, the sample size in our study was small, so speculations on the meaning of the results of this study should be cautious. Except for the correlation between the positive correlations were found between the changes Hit Rates for Go trials and changes of P3 amplitude in FC4, Fz, FCz, C4 and Cz, we did not found other correlation between the behavioral performances change and the ERP component change, which may due to relative small sample size. The sample size should be increased and female subjects should be included in the future study. Third, we only assessed one time point when we examined the effects of RS after 36 h of TSD. We are considering assessing an additional time point in order to fix this weakness of the current study. Forth, due to the absence of EEG monitoring during the recovery sleep, the results should be interpreted conservatively. Micro-sleep had not been assessed a monitored in the stage of TSD, so the effects of frequency and total duration of these micro-sleep on the results could not be estimated. Additionally, the PVT may be sensitive to TSD influence, and will be used to test the decline of alertness following TSD administration in the future study.

## Conclusion

Our results suggested that the response inhibition impairments that were induced by 36 h of TSD were improved by 8 h of RS. TSD induced a dose-dependent decrease in response inhibition that was revealed by NoGo-N2 and NoGo-P3 of prefrontal cortex activation, and 8 h of RS may help subjects to achieve a better recovery or maintain better response inhibition function. However, 8 h of RS did not result in recovery to baseline levels.

## Supporting Information

S1 File The supporting data include N2 and P3 components in NoGo trials under 5 time points in TSD and RS condition. (XLSX)
